# HIV-1 Nef Promotes Survival of Myeloid Cells by a Stat3-dependent Pathway

**DOI:** 10.1074/jbc.M103244200

**Published:** 2001-04-27

**Authors:** Scott D. Briggs, Beata Scholtz, Jean-Marc Jacque, Simon Swingler, Mario Stevenson, Thomas E. Smithgall

**Affiliations:** Department of Molecular Genetics and Biochemistry, University of Pittsburgh School of Medicine, Pittsburgh, Pennsylvania 15261; ¶ Program in Molecular Medicine, University of Massachusetts Medical Center, Worcester, Massachusetts 01605

## Abstract

Human immunodeficiency virus Nef is a small myristylated protein that plays a critical role in AIDS progression. Nef binds with high affinity to the SH3 domain of the myeloid-restricted tyrosine kinase Hck *in vitro*, identifying this Src-related kinase as a possible cellular target for Nef in macrophages. Here we show that Nef activates endogenous Hck in the granulocyte-macrophage colony-stimulating factor-dependent myeloid cell line, TF-1. Unexpectedly, Nef induced cytokine-independent TF-1 cell outgrowth and constitutive activation of the Stat3 transcription factor. Induction of survival required the Nef SH3 binding and membrane-targeting motifs and was blocked by dominant-negative Stat3 mutants. Nef also stimulated Stat3 activation in primary human macrophages, providing evidence for Stat3 as a Nef effector in a target cell for human immunodeficiency virus.

*nef* is a highly conserved gene unique to the primate lentiviruses HIV-1,^1^ HIV-2, and SIV (1, 2). Previous studies show that Nef is required for high titer replication of HIV and SIV and greatly accelerates the development of AIDS-like disease in Rhesus monkeys (3). HIV strains with defective *nef* alleles have been isolated from patients with long term, non-progressive HIV infection, implicating *nef* as a progression factor in AIDS (4, 5). Although *nef* is not required for the growth of HIV in most cultured cell lines, it has been shown to enhance HIV replication in resting peripheral blood mononuclear cells (6, 7). Furthermore, selective expression of *nef* in T-cells and monocytes/macrophages of transgenic mice is sufficient to induce a severe AIDS-like syndrome (8). However, the molecular mechanisms by which *nef* contributes to the replication and pathogenicity of HIV and SIV are not clear.

The *nef* gene encodes a 25–30-kDa myristylated protein with no identified catalytic activity and is believed to influence cellular signaling pathways by physically interacting with host cell proteins and modifying their functions (1, 2). In particular, non-receptor tyrosine kinases of the Src family have emerged as potential intracellular targets for Nef. Like Nef, Src family kinases are also myristylated and localize to cellular membranes (9). The SH3 domain of the Src-related kinase Hck binds to Nef with the highest affinity known for an SH3-mediated interaction (10, 11). Mutagenesis has established that the Nef motif responsible for SH3 binding consists of the repeated proline-rich sequence P*XX*P*XX*P*XX*P, where *X* = any amino acid (10). X-ray crystallographic and NMR structural studies of high affinity SH3-Nef complexes show that these residues form the polyproline type II helix responsible for most SH3-mediated interactions (12, 13). The Nef P*XX*P motif is highly conserved among known HIV isolates (14) and is important for SIV replication in macaques (3). These findings support the notion that the SH3 binding function of Nef and its interaction with Hck or other Src family members are essential to its functions in HIV replication and AIDS progression.

Recently, we reported that SH3-dependent interaction with Nef functionally activates full-length Hck in Rat-2 fibroblasts, leading to cellular transformation (15–17). In addition, we demonstrated that this transformation event correlates with increased cellular phosphotyrosine content and NefzHck complex formation, suggesting that Nef directly activates Hck *in vivo*. In the present study, we extend these findings by showing that Nef activates endogenous Hck in the cytokine-dependent monocyte/macrophage precursor cell line, TF-1 (18). Surprisingly, Nef produced a cytokine-independent phenotype in these cells that requires the Nef SH3 binding motif and myristylation signal sequence. Nef also induced constitutive activation of the Stat3 transcription factor in the cytokine-independent TF-1 cells, providing further evidence for the activation of endogenous Hck or a closely related tyrosine kinase by Nef. Experiments with dominant-negative Stat3 mutants indicate that Nef-induced TF-1 survival is dependent upon Stat3 activation. Nef also induced Stat3 activation in primary human macrophages. These results show for the first time that Nef can activate endogenous Src family kinases and downstream Stat factors in a cell lineage relevant to HIV infection.

## EXPERIMENTAL PROCEDURES

### Cell Culture—

The human myeloid leukemia cell line TF-1 (18) was obtained from the American Type Culture Collection (ATCC) and grown in RPMI 1640 medium supplemented with 10% fetal bovine serum, 50 *μ*g/ml gentamycin, and 3 × 10^5^ units/ml recombinant human GM-CSF (a generous gift of the Immunex Corp.). The human cell line 293T (19) was also obtained from the ATCC and grown in Dulbecco’s modified Eagle’s medium containing 5% fetal bovine serum and 50 *μ*g/ml gentamycin.

### Hck and Nef Expression Constructs—

The *nef* gene used in these studies is from the HIV-1 isolate SF2. The Nef mutants used include Nef-PA, in which Pro residues 72 and 75 were converted to Ala, and Nef-GA, in which the Gly residue at position 2 was converted to Ala. Both mutants were produced using standard polymerase chain reaction-based methods and subcloned along with wild-type Nef into the retroviral vector pSR*α*MSVtkneo (20). The resulting constructs were used to generate high titer stocks of recombinant retroviruses by co-transfection of 293T cells with an amphotropic packaging vector as described elsewhere (15–17, 21).

Construction of activated (Y501F) and kinase-dead (K269E) Hck mutants is described elsewhere (15, 22). These mutants together with wild-type Hck were subcloned into the mammalian expression vector pCDNA3 (InVitrogen) for transient expression in 293T cells. The pCDNA3-based expression vector for Stat3 has been described elsewhere (23).

The Stat3 dominant-negative mutant Stat3-EVA was supplied by Dr. Richard Jove, Moffitt Cancer Center. A point mutation converting Tyr-705 of murine Stat3 to Phe (Stat3-YF) was introduced into pcDNA3-Stat3 using the GeneEditor *in vitro* site-directed mutagenesis system (Promega). The dominant-negative Stat3 mutants were subcloned into a variant of the retroviral vector pSR*α*MSVtkneo in which the neo-antibiotic resistance marker was replaced by a DNA fragment coding for the hygromycin-resistance gene. The resulting plasmids were used to make recombinant retroviruses in 293T cells as described above.

### Soft Agar Colony Assay—

TF-1 cells were infected with recombinant Nef retroviruses using a centrifugal enhancement procedure described elsewhere (24). Briefly, TF-1 cells (5 × 10^4^) were resuspended in retroviral supernatants and centrifuged at 1,000 × *g* for 4 h in the presence of 4 *μ*g/ml Polybrene. After selection with G418 in the presence of GM-CSF, 1 × 10^4^ drug-resistant cells were plated in triplicate 35-mm dishes in RPMI 1640 medium containing 0.3% agar and 20% fetal bovine serum in the presence and absence of GM-CSF. Colony formation was assessed 2 weeks later.

### Cell Lysis and Immune Complex Kinase Assay—

TF-1 cells (10^6^) were incubated in the absence of GM-CSF for 48 h and then lysed in 1.0 ml of modified radioimmune precipitation buffer (50 mm Tris-HCl, pH 7.4, 50 mm NaCl, 1 mm EDTA, 10 mm MgCl_2_, 1% Triton X-100, 1% sodium deoxycholate, 0.1% SDS). TF-1 cell lysates were clarified by centrifugation at 100,000 × *g* for 30 min at 4 °C. Clarified cell lysates were incubated with rabbit antibodies to Hck or Fyn (Santa Cruz Biotechnology) and protein G-Sepharose (20 *μ*l of a 50% slurry) for 2 h at 4 °C. The kinase immunoprecipitates were collected by centrifugation and washed twice with 1.0 ml of radioimmune precipitation buffer followed by two washes with kinase buffer (50 mm HEPES, pH 7.4, 10 mm MgCl_2_). Kinase buffer (20 *μ*l) containing 1 *μ*g of the p50 substrate (50-kDa glutathione *S*-transferase-Sam 68 fusion protein; Santa Cruz Biotechnology) and 5 *μ*Ci [*γ*-^32^P]ATP (3,000 Ci/mmol; PerkinElmer Life Sciences) was added to the washed immunoprecipitates and incubated for 15 min at 30 °C. Reactions were quenched by adding SDS-polyacrylamide gel electrophoresis sample buffer and heating to 95 °C for 5 min. Proteins were resolved by SDS-polyacrylamide gel electrophoresis and transferred to polyvinylidene difluoride, and levels of the immunoprecipitated kinases were visualized by immunoblotting with monoclonal antibodies to Hck or Fyn (Transduction Laboratories). Radiolabeled p50 was visualized and quantitated by storage phosphor technology (Molecular Dynamics PhosphorImager).

### Electrophoretic Mobility Shift Assay—

TF-1 or 293T cells were washed once with phosphate-buffered saline containing 1 mm Na_3_VO_4_ and 5 mm NaF. Cells were resuspended with 0.5 ml of hypotonic buffer (40 mm HEPES, pH 7.9, 2 mm EDTA, 2 mm EGTA, 20 mm NaF, 1 mm Na_3_VO_4_, 1 mm Na_4_P_2_O_7_, 1 mm dithiothreitol, 0.5 mm phenylmethylsulfonyl fluoride, 50 *μ*g/ml leupeptin, 25 *μ*g/ml aprotinin, and 150 mm Na_2_MoO_4_), sonicated for 10 s, and then centrifuged for 30 s at 14,000 rpm and 4 °C. Nuclei were resuspended in high salt buffer (40 mm HEPES, pH 7.9, 840 mm NaCl, 2 mm EDTA, 2 mm EGTA, 40% glycerol, 20 mm NaF, 1 mm Na_3_VO_4_, 1 mm Na_4_P_2_O_7_, 1 mm dithiothreitol, 0.5 mm phenylmethylsulfonyl fluoride, 50 *μ*g/ml leupeptin, 25 *μ*g/ml aprotinin, and 150 mm Na_2_MoO_4_) and incubated for 30 min on a rotator at 4 °C. Nuclear extracts were clarified by microcentrifugation for 5 min at 14,000 rpm and 4 °C. The protein concentrations of the nuclear extracts were determined using the Coomassie Plus protein assay reagent (Pierce) and bovine serum albumin as standard. For the gel-shift assay, 10 *μ*g of TF-1 and 5 *μ*g of 293T nuclear extracts were used. The probe used for the Stat3 gel-shift assay is based on the *sis*-inducible element (SIE) (25). Oligonucleotides (20 pmol) were annealed in 10 *μ*l of TE buffer (10 mm Tris-HCl, pH 8.0, 1 mm EDTA) by heating to 70 °C and slowly cooling to room temperature. The probes were labeled by combining 4 *μ*l of annealed oligonucleotide with 2 *μ*l of Labeling Mix-dATP (Amersham Pharmacia Biotech), 2 *μ*l of [*γ*-^32^P]dATP (10mCi/ml; Amersham Pharmacia Biotech), and 2 *μ*l of the Klenow fragment of DNA polymerase (Life Technologies, Inc., 3.7 units/*μ*l). The mixture was incubated at room temperature for 30 min, and the unincorporated nucleotides were removed using a G-25 Sephadex spin column (Millipore). DNA binding reactions contained 40,000 cpm of labeled probe in a final volume of 20 *μ*l. Reactions were incubated at 30 °C for 30 min and run on 5% polyacrylamide gels in 0.25× Tris borate-EDTA buffer. Gels were fixed with 10% acetic acid, 10% methanol, rinsed with water, dried, and exposed to a storage phosphor screen. Images were visualized using a Molecular Dynamics PhosphorImager.

### Expression of Hck and STAT3 in 293T Cells—

Human 293T cells were transfected with Hck and Stat3 expression plasmids using a calcium phosphate-mediated transfection procedure described elsewhere (26). Cells were lysed in modified radioimmune precipitation buffer, and Stat3 expression and tyrosine phosphorylation were detected by immunoblotting with antibodies to phosphotyrosine or Stat3 (Santa Cruz Biotechnology). Control blots were performed with anti-Hck antibodies (Santa Cruz) to verify Hck expression.

### Primary Macrophage Culture, Adenovirus Infection, and Gel Shift Assay—

Monocytes were isolated by counter-current centrifugal elutriation of mononuclear leukocyte-rich cell preparations obtained from normal HIV-1 and hepatitis B seronegative donors after leukapheresis (27). Monocytes were cultured as adherent monolayers for 2 days in the presence of M-CSF (R & D Systems) and for a further 5 days in the absence of cytokine before plating. Monocyte-derived macrophages were infected 7 days after plating.

The parental adenovirus vector is a derivative of pJM17 (28) in which the entire E1- and E3-coding sequences have been deleted. The adenovirus shuttle vector (452/JL1) was constructed from pXC/JL1 (28). The HIV-1 Nef and green fluorescent protein (GFP)-coding regions were inserted in the shuttle vector under control of the cytomegalovirus immediate early promoter. The recombinant GFP and Nef adenovirus vectors were generated by homologous recombination of the parent adenovirus vector with the corresponding shuttle vector after calcium phosphate transfection of early passage 293 cells. Recombinants were propagated on 293 cells and purified through two rounds of plaque purification and concentrated by cesium chloride banding. Titers of recombinant viruses were determined by plaque formation on 293 cells (29).

To prepare nuclear extracts from macrophages, cells were washed twice with phosphate-buffered saline containing 1 mm Na_3_VO_4_ and 20 mm NaF and then lysed in buffer A (50 mm NaCl, 10 mm HEPES, pH 8.0, 500 mm sucrose, 1 mm EDTA, 0.5 mm spermidine, 0.15 mm spermine, 1 mm dithiothreitol, 0.2% Triton X-100). Nuclei were collected by centrifugation at 6500 × *g* for 3 min at 4 °C, washed in 600 *μ*l of buffer B (buffer A without Triton X-100), and collected again. Nuclear proteins were extracted at 4 °C on a rotating wheel for 30 min in buffer C (350 mm NaCl, 10 mm HEPES, pH 8.0, 25% glycerol, 0.1 mM EDTA, 0.5 mm spermidine, 0.15 mm spermine). Protease and phosphatase inhibitors were added to all buffers (20 mm NaF, 1 mm Na_3_VO_4_, 1 mm Na_4_P_2_O_7_, 1 mm phenylmethylsulfonyl fluoride, 100 *μ*M leupeptin, 0.3 *μ*M aprotinin, 10 *μ*M tosylphenylalanyl chloromethyl ketone (TPCK), 800 *μ*M 1-chloro-3-tosylamido-7-amino-2-heptanone (TLCK), 10 *μ*M bestatin, 1 *μ*M pepstatin, 10 *μ*M MG132). Extracts were clarified by centrifugation at 6500 × *g* for 5 min, and protein concentrations were determined by the Bradford assay according to the manufacturer’s instructions (Bio-Rad). For the gel shift assay, 100 ng of wild-type or mutant Stat3 double-stranded oligonucleotide probes (Santa Cruz) were end-labeled with polynucleotide kinase in the presence of 20 *μ*Ci of [*γ*-^32^P]ATP (6000Ci/mmol, PerkinElmer Life Sciences). After removal of free ATP, 1 ng of labeled probe was incubated with 2 *μ*g of nuclear proteins and 2 *μ*g of poly(dI·dC) in binding buffer (50 mm KCl, 4% glycerol, 20 mm HEPES, pH 7.9, 1 mm dithiothreitol, 5 mm MgCl_2_, 1 mm spermidine, 0.85 mm EDTA, and 0.17% Nonidet P-40) for 15 min. Bound proteins were separated on a 5% polyacrylamide gel in 0.5× Tris-borate EDTA and visualized by autoradiography.

## RESULTS

### Nef Induces Cytokine-independent Growth in the Human Myeloid Leukemia Cell Line, TF-1—

Previous work from our laboratory has shown that co-expression of Nef and Hck in Rat-2 fibroblasts induces cellular transformation (15). Hck is strongly expressed in macrophages (30–34), an important target cell for primate lentivirus infection (35, 36). To investigate whether endogenous Hck is activated by Nef in cells of the monocyte/macrophage lineage, we utilized the human myeloid leukemia cell line, TF-1 (18). This cell line is dependent on GM-CSF, IL-3, or erythropoietin for growth; removal of cytokine induces growth arrest and programmed cell death. Upon treatment with phorbol esters, TF-1 cells differentiate into macrophages.

**Fig. 1.**
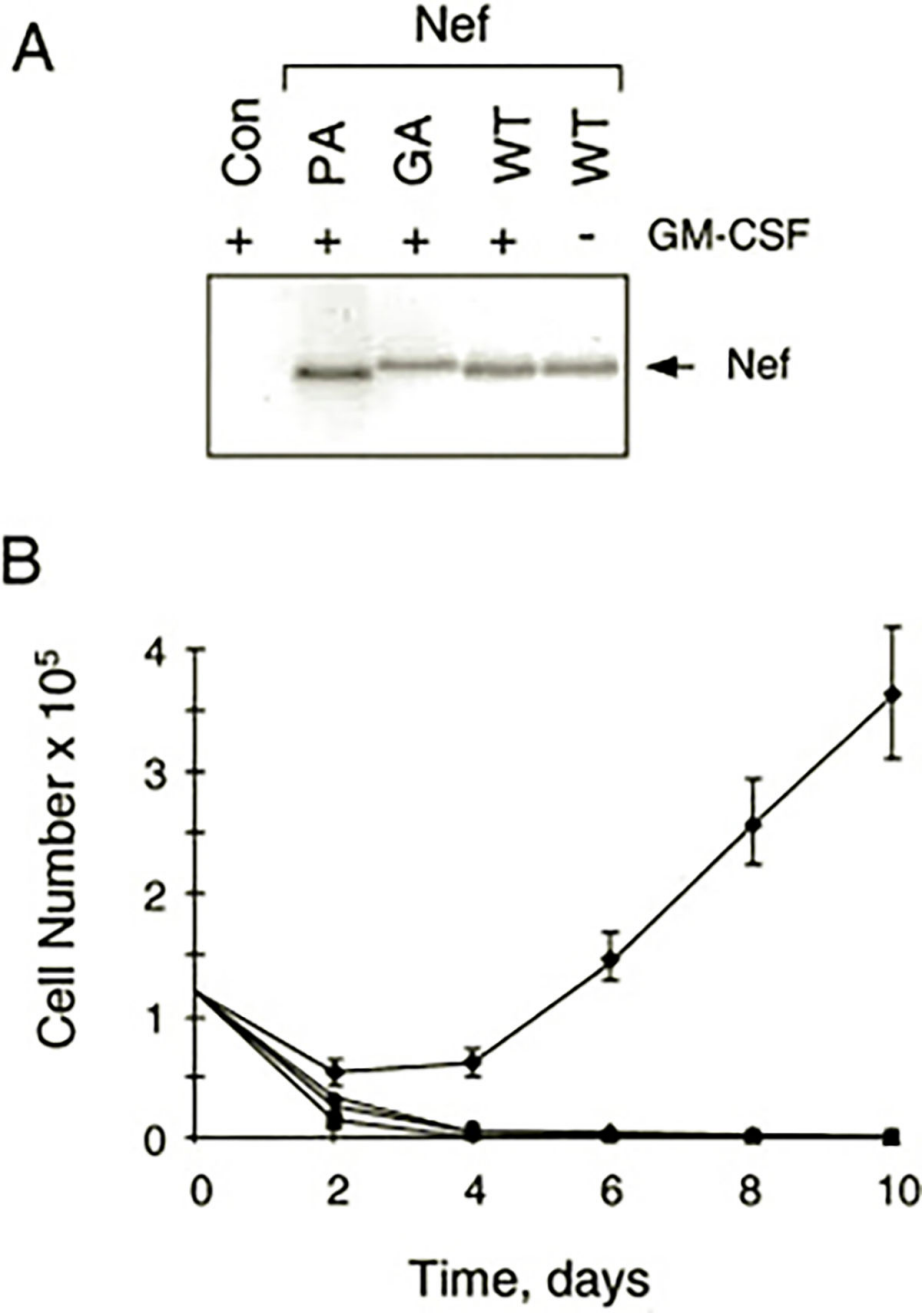
Expression of HIV-1 Nef promotes cytokine-independent outgrowth of the human GM-CSF-dependent myeloid leukemia cell line, TF-1. *A*, stable expression of Nef and Nef mutants in TF-1 cells. TF-1 cells were infected with recombinant retroviruses carrying wild-type Nef (*WT*), Nef mutants with alanine substitutions for prolines 72 and 75 in the SH3-binding motif (*PA*), or with an alanine substitution for glycine 2 in the myristylation signal sequence (*GA*). Control cells (*Con*) were infected with an empty retrovirus carrying only the *neo* selection marker. Cells were selected with G418 in the presence of GM-CSF, and Nef protein expression was verified by immunoblotting. Nef expression was also verified in TF-1 cells that emerged after infection with the wild-type Nef retrovirus and withdrawal of GM-CSF (*far right lane*). *B*, Nef promotes GM-CSF-independent outgrowth of TF-1 cells. Triplicate cultures of TF-1 cells expressing wild-type Nef (♦), Nef-PA (■), Nef-GA (▲), or control cells (●) were washed and replated at 1.25 × 10^5^ cells/ml in the absence of GM-CSF. Viable cells were counted with a hemocytometer every other day for 10 days. Results shown are the mean cell counts ± S.E.

Recombinant retroviruses were used to establish populations of TF-1 cells that stably express Nef (15, 16). After infection with Nef retroviruses, cells were placed under G418 selection in the presence or absence of GM-CSF. As shown in Fig. 1A, Nef expression was readily detected in the G418-resistant cells. To our surprise, TF-1 cells expressing Nef no longer required GM-CSF for growth (Fig. 1B). Whereas cells infected with a control retrovirus did not survive in the absence of GM-CSF, the TF-1 cells expressing Nef readily emerged from G418 selection in the absence of cytokine.

**Fig. 2.**
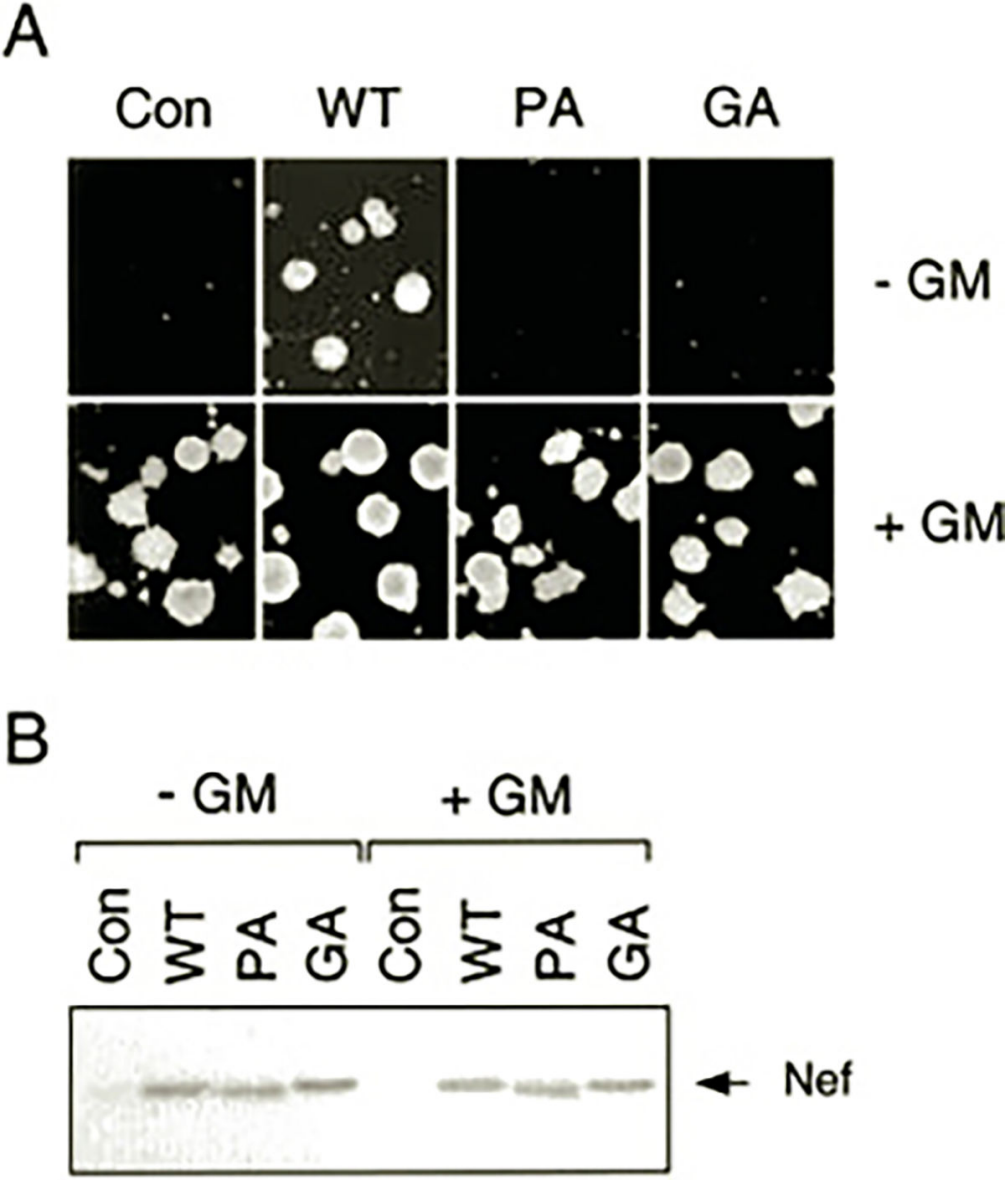
HIV-1 Nef induces cytokine-independent colony formation in TF-1 cells. *A*, human TF-1 cells were infected with recombinant retroviruses carrying wild-type Nef (*WT*) or the Nef SH3 binding (*PA*) or myristylation-defective (*GA*) mutants as described in the legend to Fig. 1. TF-1 cells were also infected with the parent retrovirus carrying only the *neo* selection marker as a negative control (*Con*). Cells were selected with G418 in the presence of GM-CSF and plated in soft agar in the presence (+) or absence (−) of GM-CSF. After incubation for 14 days, colonies were photographed under the microscope. This experiment was repeated in triplicate and revealed that nearly the same numbers of colonies were obtained with TF-1 cells expressing Nef in the presence or absence of GM-CSF (data not shown). *B*, immunoblot analysis of Nef expression of the TF-1 cultures used in *A*. Whole cell protein extracts were prepared from TF-1 cultures maintained in the presence of GM-CSF (+ *GM*, *right*) or 24 h after GM-CSF withdrawal (− *GM*, *left*). The *arrow* indicates the position of Nef on the blot.

To confirm that Nef was producing a survival signal in TF-1 cells, we also performed soft-agar colony assays. TF-1 cells were infected with recombinant Nef retroviruses, selected with G418, and plated in soft agar in the presence or absence of GM-CSF. As shown in Fig. 2A, both the parental and Nef-expressing TF-1 cells readily formed colonies in the presence of GM-CSF. However, TF-1 cells expressing Nef also formed soft agar colonies in the absence of cytokine, whereas the parental cells did not. Immunoblot analysis of protein extracts from parallel liquid cultures shows that Nef protein levels were unchanged 24 h after GM-CSF removal, indicating that Nef expression is independent of GM-CSF treatment (Fig. 2B). GM-CSF-independent outgrowth of TF-1 cells expressing Nef results from suppression of apoptosis as determined by flow cytometry of propidium iodide-stained cells as well as DNA fragmentation assays (data not shown). These results indicate that Nef is sufficient to initiate a signal transduction event in TF-1 cells permissive for survival in the absence of cytokine.

### Induction of Cytokine-independent Growth in TF-1 Cells Requires the Nef SH3 Binding Motif and Myristylation Signal Sequence—

We next investigated the structural features of Nef that are required for the induction of cytokine independence by Nef in TF-1 cells. For these experiments, we employed a Nef mutant in which the N-terminal Gly residue essential for myristylation is replaced with Ala (Nef-GA mutant). This mutation blocks Nef myristylation, which is essential for membrane targeting. We also tested a Nef mutant lacking the highly conserved P*XX*P repeat essential for interaction with the Hck SH3 domain (Nef-PA mutant). TF-1 cells expressing these Nef mutants were tested for outgrowth in liquid culture as well as colony-forming activity in soft agar in the presence or absence of GM-CSF. As shown in Figs. 1 and 2, neither of these Nef mutants was able to induce GM-CSF independence despite strong expression of the mutant proteins. These results indicate that both myristylation and the SH3 binding motif are required for cytokine independence. Thus, the TF-1 cellular target responsible for the Nef survival signal is likely to be a membrane-localized protein with an SH3 domain.

### Activation of Endogenous Hck by Nef in TF-1 Cells—

**Fig. 3.**
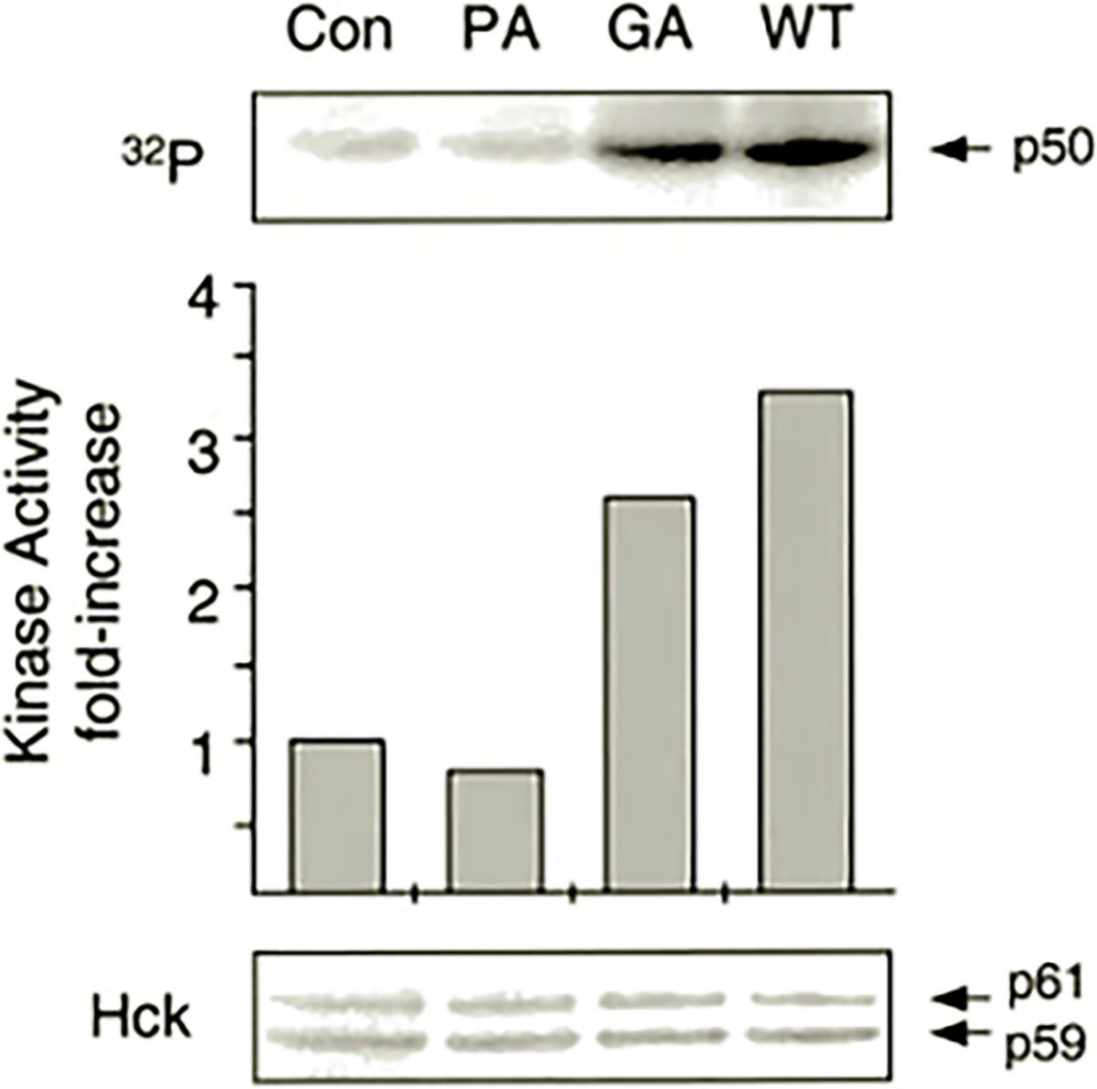
Nef activates endogenous Hck in TF-1 cells. Endogenous Hck proteins were immunoprecipitated from protein extracts of TF-1 cells stably expressing wild-type Nef (*WT*), mutant forms of Nef lacking the SH3 binding function (*PA*), or the myristylation signal sequence (*GA*) and from vector control cells (*Con*) and incubated *in vitro* with [*γ*-^32^P]ATP and a glutathione *S*-transferase-Sam 68 fusion protein of 50 kDa (p50) as substrate. After incubation, phosphorylated p50 was resolved by SDS-polyacrylamide gel electrophoresis and visualized by quantitative storage phosphorimaging (*upper panel*). The relative extent of p50 phosphorylation is shown in the *bar graph* (*middle*). The levels of the p59 and p61 forms of Hck present in each immunoprecipitate were determined by immunoblotting (*bottom*). Phosphoamino acid analysis verified the phosphorylation of p50 on tyrosine residues (data not shown).

Previous studies have shown that Hck and other members of the Src tyrosine kinase family are activated in response to GM-CSF, IL-3, and/or erythropoietin (37–41), the three cytokines known to promote survival of TF-1 cells (18). In addition, the SH3 domain of Hck binds to Nef with high affinity, leading to Hck kinase activation both *in vitro* and *in vivo* (10, 11, 15, 16, 42). These findings suggest that Nef may activate endogenous Hck in TF-1 cells as part of a signaling pathway leading to cytokine-independent growth. To determine whether Nef activates endogenous Hck, control and Nef-expressing TF-1 cells were washed free of GM-CSF and lysed, and Hck was immunoprecipitated and assayed *in vitro* with [*γ*-^32^P]ATP and a glutathione *S*-transferase-Sam 68 fusion protein (p50) as substrate. As shown in Fig. 3, TF-1 cells expressing Nef showed a greater than 3-fold increase in endogenous Hck kinase activity relative to control cells. These results suggest that activation of endogenous Hck may contribute to the survival effects of Nef in TF-1 cells. Similar levels of Hck activation have been observed after stimulation of other myeloid leukemia cell lines with IL-3 (39) or cross-linking of cell-surface Fc receptors (43). In contrast to Hck, Fyn tyrosine kinase activity was not increased by the presence of Nef in TF-1 cells (data not shown). This result is consistent with the observation that the Fyn SH3 domain binds to Nef *in vitro* with low affinity (11, 44).

We also investigated the activity of Hck in TF-1 cells expressing the Nef-PA and Nef-GA mutants. As shown in Fig. 3, Nef-PA was unable to induce endogenous Hck activation in TF-1 cells, indicating that the Nef SH3 binding motif is essential for kinase activation. This result is consistent with our previous work in Rat-2 fibroblasts co-expressing Nef and Hck (15, 16). On the other hand, mutagenesis of the myristylation signal sequence did not markedly impair the ability of Nef to induce Hck activation despite the myristylation requirement for the induction of cytokine independence by Nef. Because the Nef-GA mutant retains a functional SH3 binding motif, it is possible that this mutant interacts with the cytoplasmic pool of Hck (45, 46), resulting in kinase activation. However, activation of Hck at the plasma membrane may be required for initiation of the signaling response required for cytokine independence.

### Nef Induces Activation of Endogenous Stat3 in GM-CSF-independent TF-1 Cells—

As an alternative test for the activation of endogenous tyrosine kinase signaling in TF-1 cells by Nef, we investigated activation of the transcription factor, Stat3. Stat3 is one member of a family of transcription factors with SH2 domains that require tyrosine phosphorylation for activation (47, 48). Tyrosine phosphorylation induces Stat dimerization by reciprocal SH2-phosphotyrosine interaction, leading to nuclear translocation, DNA binding, and gene activation. Thus, Stat activation is a good indicator of endogenous tyrosine kinase activity.

**Fig. 4.**
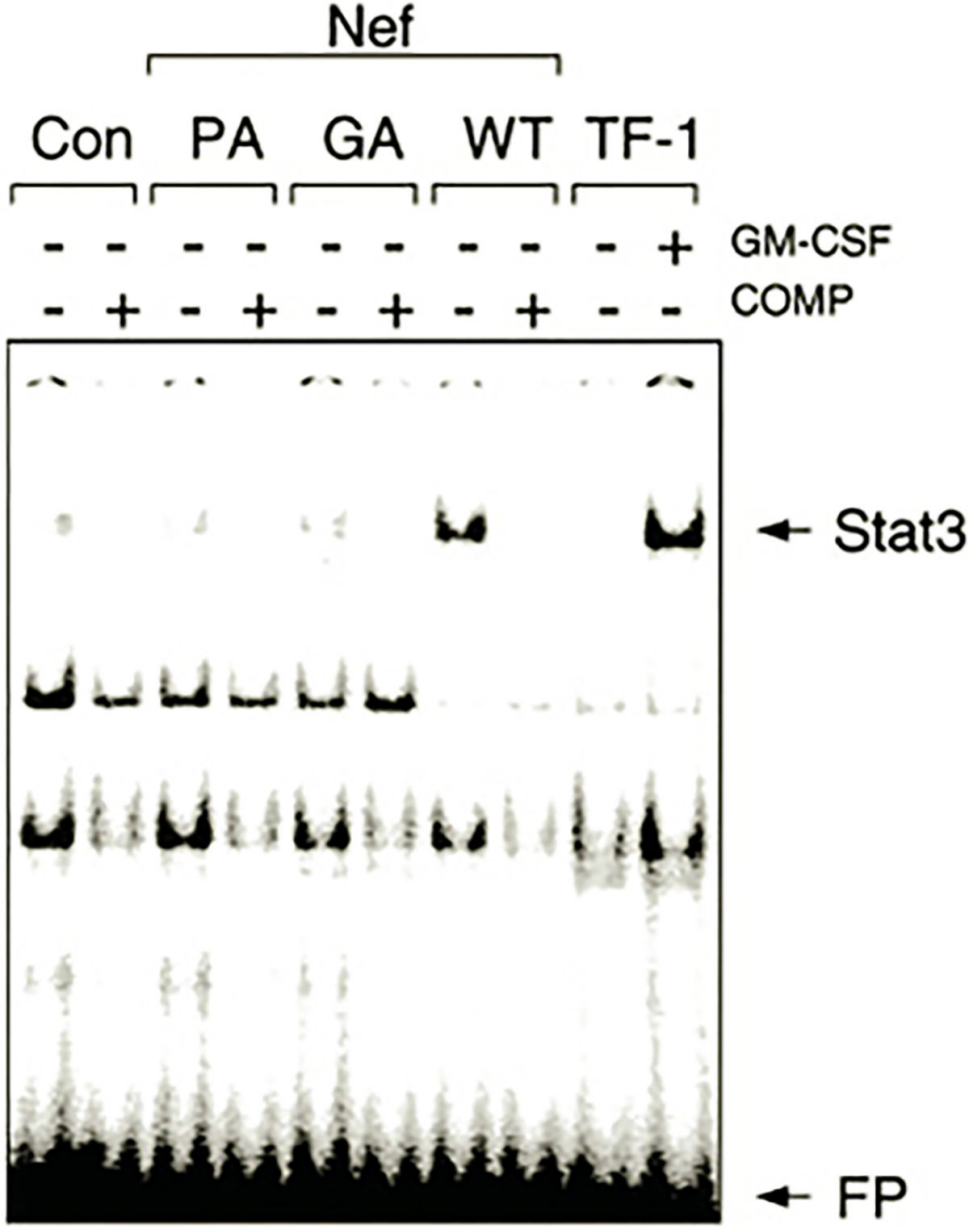
Activation of endogenous Stat3 DNA binding activity by Nef in TF-1 cells. Nuclear extracts were prepared from TF-1 cells expressing wild-type Nef (*WT*), mutant forms of Nef lacking the SH3 binding function (*PA*), or the myristylation signal sequence (*GA*) or from vector control cells (*Con*) as described under “Experimental Procedures.. Extracts were tested for the presence of activated Stat3 by gel-shift analysis with an SIE probe. To control for the specificity of DNA binding, assays were performed in the presence (+) or absence (−) of a 100-fold molar excess of unlabeled SIE oligonucleotide. As a positive control, gel-shift assays were conducted on extracts from uninfected TF-1 cells treated with (+) or without (−) GM-CSF. The positions of the shifted Stat3·SIE complex and the free probe (*FP*) are indicated by the *arrows*. Control immunoblots revealed equivalent levels of Stat3 in each of the cellular extracts (data not shown).

Stat3 activation was investigated in TF-1 cells using an electrophoretic mobility shift (gel-shift) assay with an oligonucleotide probe based on the SIE, which is potently bound by activated Stat3 (23, 25). As shown in Fig. 4, nuclear extracts prepared from the GM-CSF-independent TF-1/Nef cells produced a strong shift in SIE mobility. The gel-shifted complex was significantly reduced in control cells or in cells expressing either the SH3 binding or myristylation-defective mutants of Nef, which do not produce cytokine independence. SIE-protein complex formation was completely blocked in the presence of a 100-fold excess of unlabeled probe, indicative of specific DNA binding. No complexes were observed with a mutant SIE probe lacking nucleotides critical for Stat3 binding, and addition of anti-Stat3 antibodies to the reaction mixture completely inhibited complex formation (data not shown). Treatment of control TF-1 cells with GM-CSF produced a gel-shifted complex with electrophoretic mobility identical to that observed with the factor-independent TF-1/Nef cells, consistent with other data showing that GM-CSF induces Stat3 activation in this cell line (49). Taken together, these results demonstrate that Nef expression leads to the activation of endogenous Stat3 in TF-1 cells.

### Direct Activation of Stat3 by Hck in a Heterologous Expression System—

**Fig. 5.**
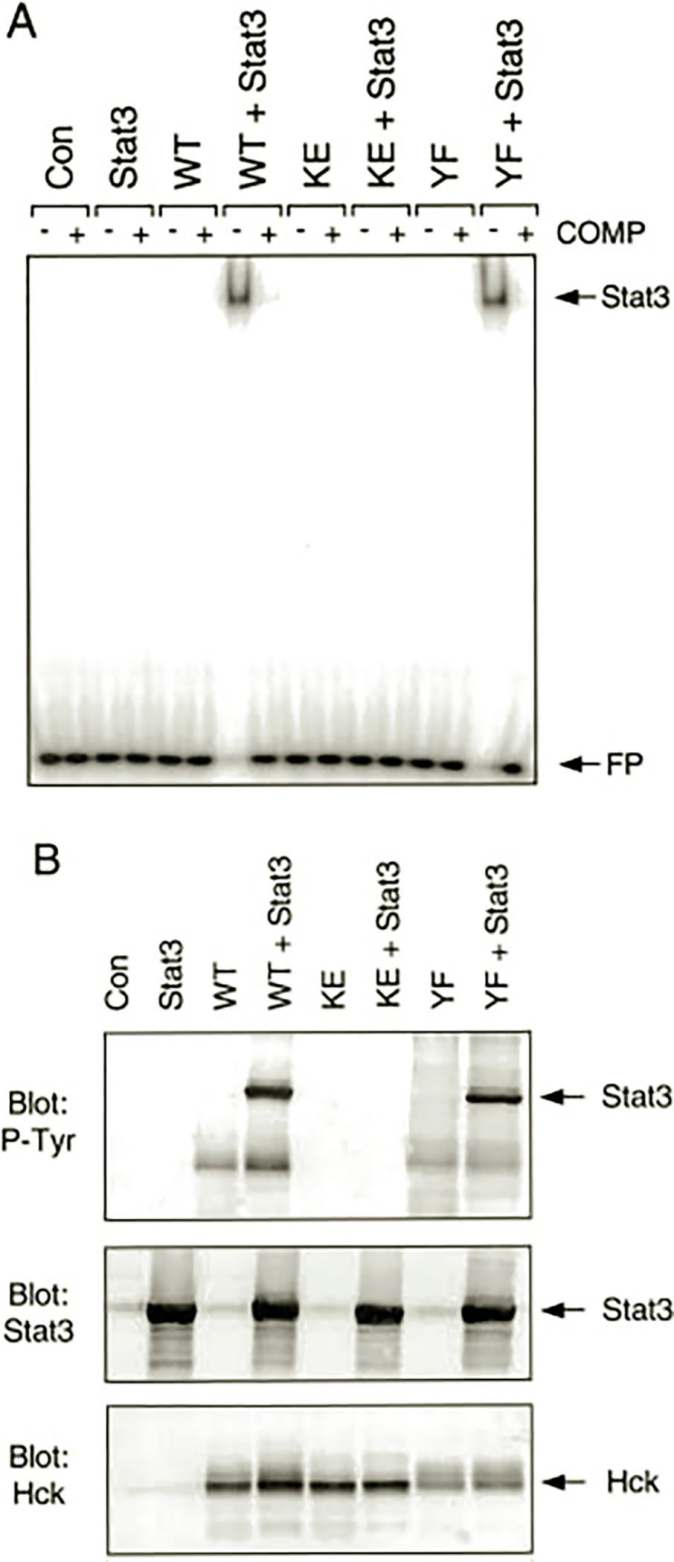
Activation of Stat3 by Hck in 293T cells. Human 293T cells were transfected with Stat3, wild-type Hck (*WT*), kinase-inactive Hck (*KE*), or a Hck mutant with a Phe substitution for the conserved tail Tyr residue (*YF*) either alone or in the combinations shown. *A*, gel-shift assay. Cytosolic extracts were prepared and tested for the presence of active Stat3 using a gel-shift assay and an SIE probe. Extracts from cells transfected with the empty vector were included as an additional negative control (*Con*). To control for the specificity of DNA binding, assays were performed in the presence (+) or absence (−) of a 100-fold molar excess of unlabeled SIE probe. The positions of the shifted Stat3·SIE complex and the free probe (*FP*) are indicated by the *arrows*. *B*, Stat3 tyrosine phosphorylation. Transfected 293T cell lysates were immunoblotted with antibodies to phosphotyrosine (*P-Tyr*, *top*), Stat3 (*middle*), or Hck (*bottom*). The *arrows* indicate the positions of Stat3 and Hck.

Data presented so far have demonstrated a strong correlation between Nef-induced cytokine independence and endogenous Hck and Stat3 activation. These results led us to speculate that Hck may be the kinase responsible for Stat activation downstream of Nef. To test this idea more directly, we expressed Hck and Stat3 either alone or together in 293T cells and assayed Stat3 activation using the gel-shift assay. As shown in Fig. 5A, co-expression of Hck and Stat3 in 293T cells produced a dramatic shift in probe mobility, indicative of potent Stat3 activation. In contrast, expression of either protein alone was without effect, consistent with the direct activation of Stat3 by Hck in this system. As an additional negative control, we co-expressed Stat3 with a kinase-inactive mutant of Hck in this system. No gel-shift response was detected, demonstrating the requirement for tyrosine phosphorylation in the activation mechanism. Finally, we also tested a transforming variant of Hck in which the negative regulatory tyrosine residue in the C-terminal tail (Tyr-501) has been converted to Phe (15). This Hck mutant activated Stat3 to the same extent as wild type, suggesting that 293T cells lack sufficient Csk for effective negative regulation of wild-type Hck. Csk is the kinase responsible for the negative regulation of Src family kinases and works by phosphorylating the conserved tyrosine residue in Src family kinase C-terminal tails. As a result, the phosphorylated tail engages the SH2 domain in an intramolecular fashion, contributing to kinase down-regulation.

To establish that Stat3 DNA binding activity induced by Hck correlated with Stat3 tyrosine phosphorylation, lysates from the transfected 293T cells were immunoblotted with anti-phosphotyrosine antibodies. Fig. 5B shows that Stat3 is strongly phosphorylated on tyrosine when co-expressed with either of the active forms of Hck, consistent with the gel-shift result. In contrast, lysates from cells expressing Stat3 alone or in the presence of the kinase-inactive form of Hck showed no evidence of Stat3 tyrosine phosphorylation. Control immunoblots show that approximately equivalent levels of Stat3 and Hck proteins were expressed. These results provide strong evidence that Hck can induce direct activation of Stat3 under defined conditions and suggest that Nef may trigger Stat3 activation via Hck in TF-1 cells.

### Activation of Stat3 Is Required for Nef-induced Survival of TF-1 Cells—

**Fig. 6.**
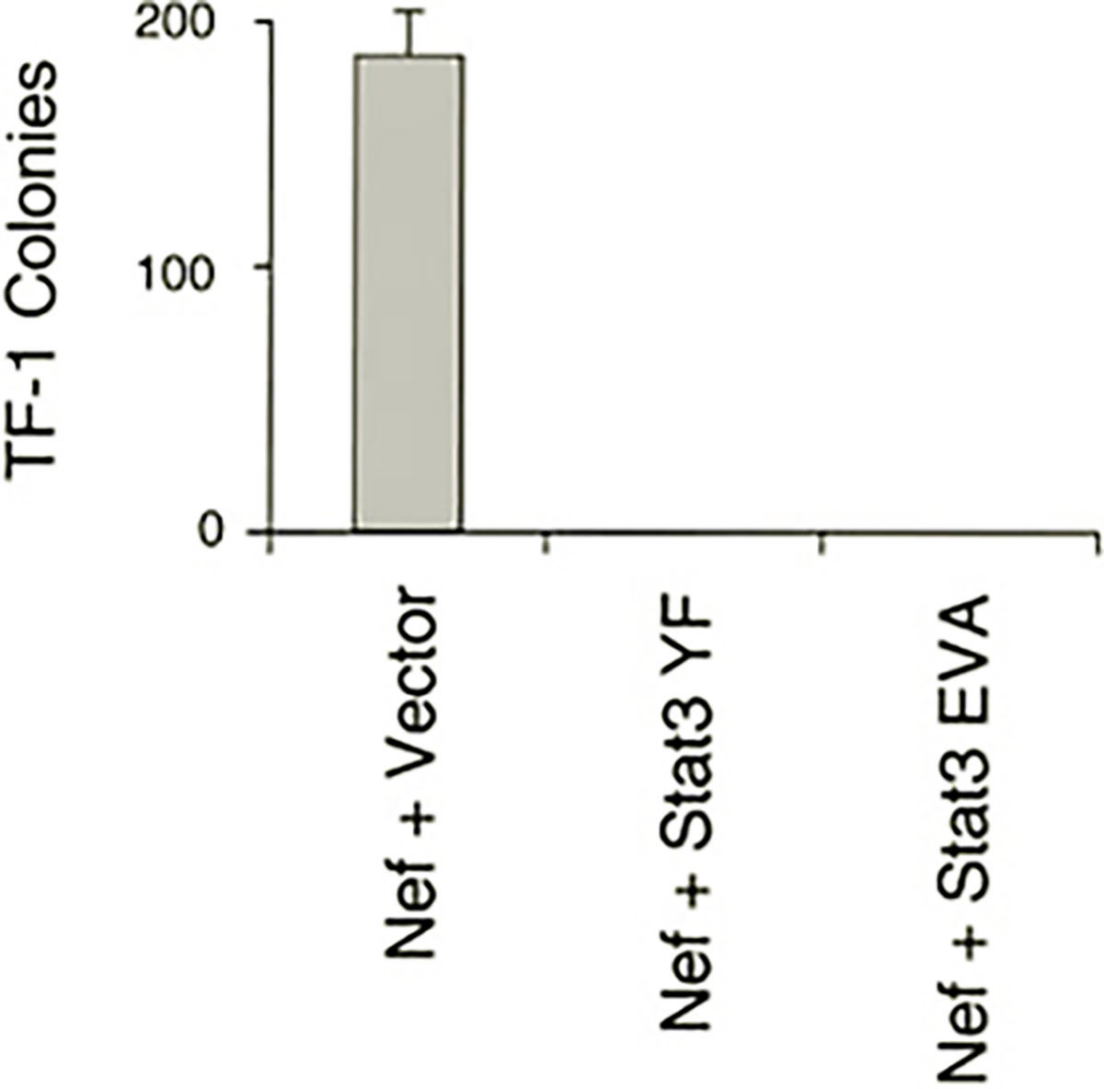
Suppression of Nef-induced TF-1 cell colony formation by dominant-negative mutants of Stat3. TF-1 cells were infected with recombinant retroviruses carrying Stat3 mutants lacking either the tyrosine phosphorylation site (*Stat3 YF*) or glutamic acid residues essential for DNA binding (*Stat3 EVA*). As a control, cells were infected with a virus carrying the hygromycin selection marker alone (*Vector*). Twenty-four hours later, the cells were superinfected with the Nef retrovirus (linked to the *neo* selection marker) and selected with hygromycin and G418 in the presence of GM-CSF. Drug-resistant cells were plated in soft agar in the presence of hygromycin and G418 and in the absence of GM-CSF. Macroscopic colonies were stained and counted after 14 days. The experiment was plated in triplicate, and the average number of colonies is shown ±S.E.

To determine whether activation of Stat3 is necessary for Nef-induced cell survival, we employed two dominant-negative mutants of Stat3. The first mutant (YF) lacks the site of tyrosine phosphorylation (Tyr-705), whereas the second (EVA) has mutations in Glu residues required for DNA binding. These and related Stat3 mutants have been used to block Stat3-mediated biological responses in other systems (50–52) including transformation of fibroblasts by v-Src (53, 54). TF-1 cells were co-infected with recombinant retroviruses carrying Nef and the dominant-negative Stat3 mutants. Nef and the Stat3 mutants were linked to different drug selection markers (neo and hygromycin, respectively) to ensure that cells were infected with both retroviruses after double selection. Drug-resistant cells were then plated in soft agar and scored for colony outgrowth in the absence of GM-CSF. Nef readily induced colony formation in cells that were also infected with a control retrovirus carrying only the hygromycin resistance marker (Fig. 6). However, when cells were doubly infected with Nef and either of the Stat3 dominant-negative mutants, no colony outgrowth was observed. These data suggest that activation of Stat3 is an essential part of the survival response induced by Nef in TF-1 cells.

### Nef Activates Stat3 in Primary Human Macrophages—

**Fig. 7.**
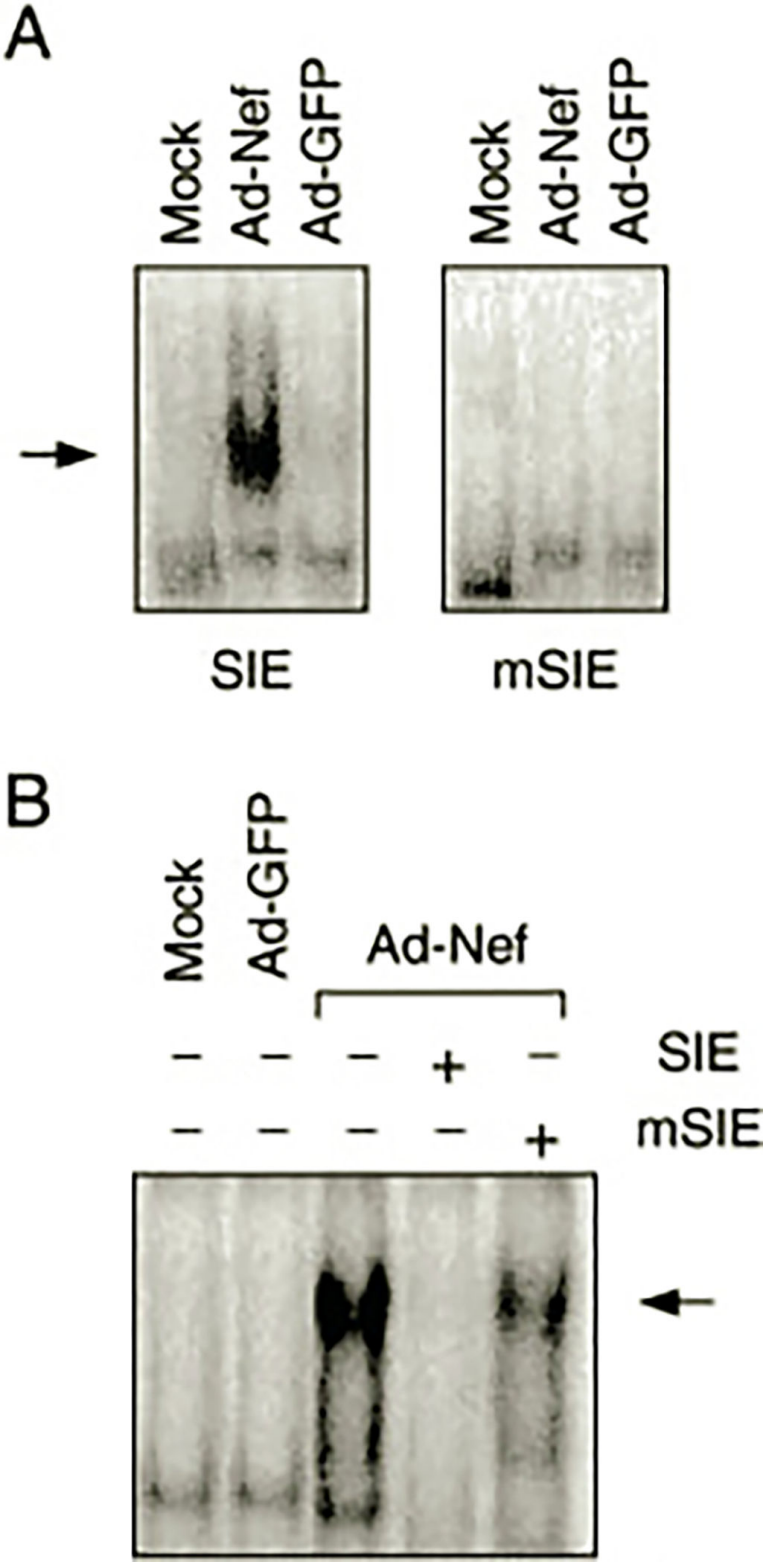
Nef activates endogenous Stat3 in human macrophages. Primary cultures of macrophages were infected with recombinant adenovirus vectors carrying Nef (*Ad-Nef*) or green fluorescent protein (*Ad-GFP*) cDNAs. Mock-infected cells were also included as an additional negative control. *A*, nuclear extracts were prepared and tested for the presence of activated Stat3 by gel-shift analysis with wild-type or mutant (*m*) SIE probes. *B*, to control for specificity in the gel-shifted complexes, duplicate assays were conducted in the presence (+) of an excess of unlabeled wild-type or mutant SIE probes. The *arrows* indicate the presence of the gel-shifted complexes.

A final series of experiments investigated whether Nef can induce Stat3 activation in primary human monocyte-derived macrophages, a target cell for HIV infection. For these experiments, recombinant adenovirus vectors were used to introduce Nef into primary cultures of macrophages isolated from normal donors. Parallel cultures were infected with a control adenovirus carrying GFP. Nuclear protein extracts were prepared from Nef-infected and control macrophages and analyzed for the presence of activated Stat3 using the SIE probe. As shown in Fig. 7A, extracts from macrophages infected with the Nef adenovirus produced a marked shift in SIE mobility, whereas extracts from control cells infected with the GFP adenovirus or mock-infected cells had no effect. Duplicate gel shift assays using an SIE probe with mutations in the sequence required for Stat3 binding produced no detectable protein-DNA complexes. Similarly, the addition of an excess of unlabeled SIE probe to the reaction completely inhibited formation of the gel-shifted complex, whereas the mutant SIE did not (Fig. 7B). These results are consistent with the observations made in TF-1 cells after expression of Nef (Fig. 4) and provide direct evidence that Nef expression is sufficient to activate both tyrosine kinase activity and Stat3 in primary macrophages.

## DISCUSSION

Previous work from our laboratory established that SH3-mediated interaction of Nef with Hck is sufficient to induce oncogenic transformation when these proteins are co-expressed in Rat-2 fibroblasts (15–17). This result suggested that Nef may constitutively induce Hck activation in target cells for HIV that express this Src-related kinase, such as macrophages. In this study, we provide evidence that Nef can induce endogenous Hck activation in the monocyte/macrophage precursor cell line, TF-1. Expression of Nef in TF-1 cells also revealed a new biological property of Nef: its ability to induce cytokine-independent myeloid cell growth. This finding suggests that Nef is able to stimulate some of the signal transduction pathways normally activated by GM-CSF that lead to survival and proliferation in this cell line. Several observations suggest that activation of Hck may contribute to the effect of Nef on TF-1 cell survival. First, Nef-induced activation of Hck (Fig. 3) but not Fyn (data not shown) in the factor-independent cells correlates with the binding affinities of Nef for the SH3 domains of these Src family kinases (11, 44). Second, the Nef mutant lacking the polyproline motif responsible for SH3 binding does not produce the cytokine-independent phenotype in TF-1 cells (Figs. 1 and 2) and is unable to activate endogenous Hck in these cells (Fig. 3). In previous work, we and others show that the Nef SH3 binding motif is essential for interaction with Hck and subsequent kinase activation (10, 15). Finally, Hck is normally activated downstream of cytokine receptors, including those for GM-CSF and IL-3 (38–40). Both of these cytokines have been shown to promote growth and survival of TF-1 cells (18).

Previous work has established that Nef may mimic the effect of IL-2 on T-lymphocyte activation (55). This study employed a monkey T-lymphoid cell line, known as 221, which requires IL-2 to proliferate. SIV was found to replicate efficiently in IL-2-stimulated 221 cells independently of the presence of Nef. However, *nef*+ viruses replicated as much as 100-fold more efficiently than *nef*-defective viruses in the absence of IL-2. This difference was attributed to the ability of Nef to induce IL-2 production in the host cell line. These results suggest that our observation of Nef-induced survival in myeloid cells may also involve an autocrine mechanism, possibly through the induction of GM-CSF, IL-3, or other cytokines known to promote TF-1 cell survival. However, we found that culture medium conditioned by cytokine-independent TF-1/Nef cells was unable to support naive TF-1 cell growth (data not shown). Thus, Nef appears to promote TF-1 cell survival via constitutive stimulation of a host survival pathway rather than through the induction of anti-apoptotic cytokines.

The observation that Nef-induced activation of Hck correlates with cytokine-independent survival of TF-1 cells suggests that activation of Hck alone may be sufficient to induce the survival response. We have tested this possibility by expressing two different constitutively activated forms of Hck in TF-1 cells using recombinant retroviruses. The first of these was activated by Phe substitution of the conserved Tyr residue in the C-terminal tail, whereas the second has mutations of two proline residues in the SH2-kinase linker region that interact intramolecularly with the SH3 domain in the inactive form of the kinase (15, 16). Although both of these mutants are strongly transforming in Rat-2 fibroblasts, neither was able to produce cytokine-independent outgrowth of TF-1 cells. This result suggests that activation of Hck alone may not be sufficient to produce the survival signal. Nef may activate other members of the Src kinase family, which could in turn contribute to Stat activation and the observed biological effect. One possibility is Lyn, which has been shown to mediate the GM-CSF signal for survival in neutrophils (56). The Lyn SH3 domain has been shown to interact with Nef *in vitro* (10), suggesting that Nef may induce Lyn activation in a manner analogous to Hck. However, we have observed that co-expression of Lyn with Nef in fibroblasts does not induce Lyn activation under conditions where Hck is potently activated by Nef (17). This result argues against a contribution of Lyn to the observed survival signal produced by Nef in TF-1 cells. Alternatively, Nef-mediated activation of serine/threonine kinases may be required for full transcriptional activation of Stat3 (57). Nef has been recently shown to activate the c-Jun N-terminal kinase (Jnk) downstream of Nak (58), a Pak-related kinase that interacts directly with Nef (59, 60). Interestingly, Jnk and the related p38 kinase have recently been identified as the activities responsible for serine phosphorylation and transcriptional activation of Stat3 in v-Src-transformed fibroblasts (61). Thus it is possible that Nef may activate multiple kinase signals that are convergent on Stat3.

Data presented here show for the first time that expression of HIV Nef is sufficient to activate a Stat signal transduction pathway. Data in Fig. 4 demonstrate that Nef activates Stat3 DNA binding activity to the same extent as GM-CSF treatment in TF-1 cells. Stat3 activation is required for the generation of survival signals by Nef, as two different dominant-negative Stat3 mutants were able to interfere with cytokine-independent colony formation (Fig. 6). This result agrees with the work of Fukada *et al.* (50), who demonstrate that Stat3 dominant-negative mutants were able to interfere with survival signals generated by gp130, the signal-transducing receptor subunit shared by the IL-6 family of cytokines. Hck has been implicated in leukemia inhibitory factor signal transduction, which is linked to gp130 (62, 63). Data presented in Fig. 5 show that Hck can directly induce Stat3 DNA binding activity and tyrosine phosphorylation in a defined expression system (293T cells). These results are consistent with the possibility that activation of Hck by Nef may be sufficient to induce Stat3 tyrosine phosphorylation in TF-1 cells. In addition, a recent study has shown that Src kinases, and not Jaks, are responsible for Stat activation in response to IL-3 treatment in some cases (64). More recent work has shown that constitutive activation of Stat3 suppresses apoptosis in human multiple myeloma cells (65). This study also demonstrated the Stat3-dependent induction of the Bcl-2 family member Bcl-X_L_ as a possible mechanism for the observed anti-apoptotic effects of Stat3. Whether a similar mechanism accounts for the Nef-induced survival of TF-1 cells will require further investigation.

In addition to the TF-1 cell line, we observed that Nef expression is sufficient to activate Stat3 in primary human macrophages, raising the possibility that Nef may mimic cytokine signaling in this target cell for HIV. Although the biological significance of this effect is currently unknown, it is reasonable to speculate that Nef may contribute to macrophage survival by a mechanism similar to that observed with TF-1 cells. Interestingly, HIV-1 infection of primary human macrophages promotes their survival in culture.^2^ Because Hck is a myeloid-restricted tyrosine kinase, constitutive activation of a Hck-Stat3 pathway by Nef may promote virus replication and dissemination. Recently, it was demonstrated that monocytotropism promotes amplification and spread of SIV in macaques (36). This finding raises the possibility that Nef modifies the physiology of macrophages in a way that promotes their role as persistent viral reservoirs. Our observation that Nef may serve as a survival factor is consistent with this role. The identification of cellular pathways through which Nef mediates survival and other effects may shed further light on the role of this accessory gene product in virus replication and pathogenicity.

## Footnotes

1 The abbreviations used are: HIV human immunodeficiency virus; GM-CSF, granulocyte-macrophage colony-stimulating factor; SIV, simian immunodeficiency virus; GFP, green fluorescent protein; IL, interleukin; SIE, *sis*-inducible element.

2 B. Brichacek and M. Stevenson, unpublished observation.
